# Inhibition of candidalysin production by methoxy-apo-enterobactin from *Streptomyces ambofaciens* CJD34 as a novel antifungal strategy against *Candida albicans*

**DOI:** 10.71150/jm.2504019

**Published:** 2025-06-30

**Authors:** Eui-Seong Kim, Hyeongju Jeong, Mustansir Abbas, Soohyun Um, Juntack Oh, Kyuho Moon, Kyung-Tae Lee

**Affiliations:** 1 Korea Zoonosis Research Institute, Jeonbuk National University, Iksan 54531, Republic of Korea; 2 College of Pharmacy, Kyung Hee University, Seoul 02447, Republic of Korea; 3 Department of Biomedical and Pharmaceutical Sciences, Graduate School, Kyung Hee University, Seoul 02447, Republic of Korea; 4 College of Pharmacy, Yonsei University, Incheon 21983, Republic of Korea; 5 Department of Regulatory Science, Graduate School, Kyung Hee University, Seoul 02447, Republic of Korea; 6 Institute of Regulatory Innovation through Science, Kyung Hee University, Seoul 02447, Republic of Korea

**Keywords:** *Candida albicans*, candidalysin, antivirulence agent, methoxy-apo-enterobactin

## Abstract

Opportunistic fungal pathogens, responsible for over 300 million severe cases and 1.5 million deaths annually, pose a serious global health threat, especially in immunocompromised individuals. Among these, *Candida albicans* is a major cause of both superficial and invasive infections, which can progress to systemic candidiasis. One of the critical factors in *C. albicans* pathogenicity is the yeast-to-hyphal transition, which enables biofilm formation and promotes tissue invasion through the secretion of candidalysin, a cytolytic peptide toxin encoded by the *ECE1* gene. In this study, metabolites produced by *Streptomyces ambofaciens* CJD34, isolated from soil samples, were screened for antifungal activity. Methoxy-apo-enterobactin (compound 1) was identified as a potential inhibitor of *C. albicans* virulence. Treatment with compound 1 significantly suppressed *ECE1* expression and candidalysin production. In a murine subcutaneous infection model, topical application of compound 1 reduced subcutaneous colonization by *C. albicans*. Molecular docking analysis suggested that the inhibition of *ECE1* expression was not mediated by direct binding to known upstream transcription factors, indicating an indirect mechanism of action. Collectively, these findings highlight compound 1 as a promising antivirulence agent targeting candidalysin-mediated pathogenicity in *C. albicans*.

## Introduction

Opportunistic fungal pathogens are responsible for over 300 million severe cases and 1.5 million deaths annually worldwide, either as primary infections or as complications that exacerbate underlying diseases (Zhang et al., 2023). Fungal infections can range from superficial to deeply invasive forms, and in immunocompromised individuals, systemic infections can become life-threatening (Brown et al., 2012).

Candidiasis, primarily caused by *Candida albicans*, can extend beyond the skin and mucosal tissues to affect multiple organ systems. This opportunistic infection is particularly dangerous and has been classified among the critical fungal priority pathogens by the World Health Organization (WHO). Interestingly, in healthy individuals, *C. albicans* exists as a commensal organism within the gastrointestinal tract, genitals, oral cavity, and skin. However, when the immune system is impaired, *C. albicans* can transition from a benign commensal to an invasive pathogen. Upon entering the bloodstream, it can disseminate to vital organs including the cardiovascular system, central nervous system, kidneys, spleen, liver, eyes, and bones, ultimately leading to systemic candidiasis (Zhang et al., 2024).

One of the key factors in tissue invasion by *C. albicans* is the yeast-to-hyphal transition, followed by the formation of a biofilm. Biofilm development proceeds through a sequential process involving initial cell attachment, hyphal transition, and extracellular matrix secretion. Candidalysin, an epithelial-damaging peptide toxin, is secreted after the hyphal transition. The *ECE1* gene encodes a preproprotein Ece1, which undergoes a two-step posttranslational processing mediated by the proteinases Kex2 and Kex1. This processing results in the secretion of candidalysin, a 31-amino acid polypeptide (Moyes et al., 2016; Richardson et al., 2018). Given the central role of candidalysin in pathogenesis, there is a need for antifungal agents that specifically target candidalysin production or function.

In this study, metabolites produced by *Streptomyces ambofaciens* CJD34, isolated from soil samples, were separated and identified. Among them, compounds exhibiting antifungal activity against *C. albicans* were distinguished. Methoxy-apo-enterobactin (compound 1) isolated from CJD34 was found to specifically inhibit the expression of the *ECE1* gene, which encodes candidalysin, a key virulence factor. In a murine subcutaneous infection models, topical administration of compound 1 tended to reduce the *C. albicans* colonization. Structural analysis suggests that inhibition of *ECE1* expression is not due to direct binding of compound 1 to upstream transcription factors, indicating the involvement of an indirect regulatory mechanism. These findings highlight the potential of compound 1 as an antivirulence agent targeting candidalysin production in *C. albicans*. However, given the limited research on the antifungal potential of *S. ambofaciens*-derived metabolites, this study provides novel insights into the development of new antifungal strategies.

## Materials and Methods

### General experimental procedures for metabolite analysis

Optical rotations were measured by a Jasco P-2000 polarimeter (Japan) with a 1.0-cm cell. UV spectra and liquid chromatography-mass spectrometry (LC/MS) data were recorded using an Agilent G6125B MSD system coupled with an Agilent Technologies 1260 series Infinity II LC system using a reversed-phase C18 column (Phenomenex Luna, 100 × 4.6 mm, 5 µm). Infrared (IR) spectra were acquired using a Spectrum 3 Fourier Transform Infrared Spectrometer (PerkinElmer, USA). High-resolution electrospray ionization mass spectra (HR-ESI-MS) were obtained using an Agilent Technologies 1290 series HPLC coupled with an Agilent 6530 iFunnel Q-TOF LC/MS system (Agilent Technologies, USA). ^1^H, ^13^C, and 2D NMR spectra were recorded on an Agilent VNMRS 600 MHz at the Korea Basic Science Institute (KBSI) in Gwangju and a Bruker Advance III 700 MHz and a Bruker Advance II 900 MHz spectrometer at the KBSI in Ochang. HPLC purification was performed on a Waters system (1525 binary HPLC pump and 996 photodiode array detector) with a YMC Pack-ODS-A-C18 column (250 ×10 mm, 5 μm).

### Bacterial isolation and identification

Soil samples were collected from Dongbaekdongsan Wetland in the Gotjawal Forest on Jeju Island, Republic of Korea. Mixtures of dried samples and 4 ml of sterilized water (2 g each) were heated to 55°C and sonicated. The mixtures were spread on A1 medium (agar 18 g, and 1 L of distilled water), Chitin medium (chitin 4 g, K_2_HPO_4_ 0.75 g, MgSO_4_∙7H_2_O 0.5 g, KH_2_PO_4_ 3.5 g, FeSO_4_∙7H_2_O 10 mg, MnCl_2_∙7H_2_O 10 mg, ZnSO_4_∙7H_2_O 10 mg, agar 18 g, and 1 L of distilled water), *Streptomyces* isolation medium (casein 0.4 g, starch 1 g, KNO_3_ 0.5 g, K_2_HPO_4_ 0.2 g, MgSO_4_∙7H_2_O 0.1 g, CaCO_3_ 0.1 g, agar 18 g, and 1 L of distilled water), tap water-yeast extract medium (K_2_HPO_4_ 0.5 g, yeast extract 0.25 g, agar 18 g, and 1 L of tap water), Kuster medium (glycerol 10 ml, casein 0.3 g, KNO_3_ 3 g, K_2_HPO_4_ 2 g, NaCl 2 g, MgSO_4_∙7H_2_O 0.05 g, CaCO_3_ 20 mg, FeSO_4_ 10 mg, agar 18 g, and 1 L of distilled water) with cycloheximide (50 mg/L) and nalidixic acid (20 mg/L). The bacterial strain CJD34 was isolated from the Chitin agar medium of the Gotjawal sample and was identified as *Streptomyces* sp. (98.5% identical to *Streptomyces ambofaciens*) based on the 16S rDNA gene sequence analysis (GenBank accession number CP012382.1).

### Cultivation and extraction

The CJD34 strain was cultured in 50 ml of yeast extract-malt extract medium (glucose 10 g, yeast extract 3 g, malt extract 3 g, peptone 5 g, soybean extract 2 g, and 1 L of distilled water) in 125 ml flask on a rotary shaker for 3 days at 30°C and 190 rpm. For first scale-up, a 3.5 ml aliquot of the broth culture was used to inoculate 150 ml of YEME medium in a 500 ml flask, which was then cultured under the same conditions. 20 ml of the culture was transferred to 1 L of YEME medium in 2.5 L ultra-yield flasks (total volume 10 L) for 5 days at the same fermentation conditions. The CJD34 strain was isolated with 15 L of ethyl acetate from a whole culture (EtOAc). The EtOAc and water layers were separated, and residual water in the EtOAc layer was eliminated by adding anhydrous sodium sulfate. The EtOAc extract was concentrated under vacuum to produce the solid substance.

### Isolation and purification of compounds

The CJD34 strain dried material was fractionated over a C18 reversed-phase column (YMC ODS-A C18, 50 μm silica gel) using a step gradient solvent system (20, 40, 60, 80, and 100% MeOH/H_2_O), respectively. Compound 1, 2, and 3 were found in the CJD34 60% MeOH/H_2_O fraction. Each fraction was filtered with a syringe filter (Advantec, HP020AN) and then subjected to semi-preparative reversed-phase HPLC with a flow rate of 2 ml/min using a linear gradient condition and an isocratic solvent system. CJD34 60% fraction was purified under isocratic 30% CH_3_CN/H_2_O (YMC ODS-A-C18: 250 × 10 mm, 5 µm, UV detection at 254 nm). Compound 1 was eluted at 34.4 min, compound 2 at 23.4 min, and compound 3 at 32.7 min. Finally, the samples were further purified to yield pure compounds 1 (4.8 mg), 2 (7 mg), and 3 (22 mg).

Methoxy-apo-enterobactin (1): brown oil, $[\alpha {]}_{D}^{25}$+5.54 (*c* 0.1, MeOH); UV (MeOH) λ_max_ (log ε) 225 (4.80) nm, 250 (4.41) nm 315 (3.90); IR (neat) ν_max_ 3350, 2957, 1738, 1536, 1275, 1186, 1077 cm^-1^, ^1^H and ^13^C NMR data see Table 1, HR-ESI-MS *m/z*: [M+H]^+^ 702.1769 (calcd for C_31_H_32_N_3_O_16_, 702.1777)

*N*-(2,3-dihydroxybenzoyl)-L-serine3-[2-[(2,3-dihydroxybenzoyl)amino]-2-propenoate] (2): brown oil, $[\alpha {]}_{D}^{25}$ +6.14 (*c* 0.1, MeOH); UV (MeOH) λ_max_ (log ε) 225 (4.54) nm, 250 (4.14) nm 315 (3.73); IR (neat) ν_max_ 3412, 2887, 1756, 1673, 1464, 1366, 1275 cm^-1^, ^1^H and ^13^C NMR data see Table 1, HR-ESI-MS *m/z*: [M+H]^+^ 447.1019 (calcd for C_20_H_19_N_2_O_10_, 447.1034)

*N*, *N*′, *N*′′-Tris(2,3-dihydroxybenzoyl)-*O*-(*α*-aminoacryloyl)-*O*-serylserine (3): brown oil, $[\alpha {]}_{D}^{25}$+1.54 (*c* 0.1, MeOH); UV (MeOH) λ_max_ (log ε) 225 (4.79) nm, 250 (4.31) nm 315 (3.84); IR (neat) ν_max_ 3443, 2950, 1739, 1646, 1460, 1342, 1236 cm^-1^, ^1^H and ^13^C NMR data see Table 1, HR-ESI-MS *m/z*: [M+H]^+^ 670.1494 (calcd for C_30_H_28_N_3_O_15_, 670.1514)

### Determination of absolute configurations of amino acids by advanced Marfey's method

The absolute configurations of the 1, 2, and 3 were determined using advanced Marfey’s method. After hydrolyzing 1 mg of 1 in 0.5 ml of 6 N HCl at 120°C for 30 min, the reaction was cooled rapidly by immersing the reaction vial into cold water for 10 min. After evaporating the reaction solvent three times under a vacuum, any remaining hydrochloric acid was neutralized by adding 0.5 ml of water and repeating the process. After drying the hydrolysate, it was lyophilized for 2 h. The hydrolysate of 1 was separated into two equal portions, and each portion was thereafter placed in a separate vial containing 8 ml. After that, the hydrolysate of 1 were dissolved in 100 μl of 1 N sodium hydroxide. 50 μl of either 10 mg/ml L-FDLA (1-fluoro-2,4-dinitrophenyl-5-L-leucine amide) or D-FDLA (1-fluoro-2,4-dinitrophenyl-5-D-leucine amide) in acetone was added to each of the two vials. After incubating the vials at 80°C, the reaction mixtures were analyzed. To stop the reaction, an aliquot of 2 N HCl containing 50 μl was added to the vial, and then 300 μl of an aqueous solution containing 50% CH_3_CN was added. An aliquot of each reaction mixture that was 5 μl in volume was subjected to LC-MS analysis using a gradient solvent system that consisted of 20% to 60% CH_3_CN containing 0.1% formic acid over 50 min. Other compounds 2 and 3 underwent the same process. In the hydrolysate of 1, the L-FDLA derivative was found to elute before the D-FDLA derivative. As a result, the absolute configurations of 1, 2, and 3 were determined to be L.

### Animal ethics statement

Animal care and research were approved after deliberation by the Institutional Animal Care and Use Committee of the Experimental Animal Center at Jeonbuk National University (Approval number JBNU 2023–131). All experiments followed the experimental ethics guidelines.

### Fungal strains and growth conditions

*C. albicans* SC5314, *Candida auris* B8441, and *Cryptococcus neoformans* H99 strains were grown at 35°C incubator, and sub-cultured in yeast-extracted peptone dextrose (YPD) broth to perform the experiments.

### Minimal Inhibitory Concentration (MIC) assay

We conducted MIC assays to determine the efficacy of compounds against *C. albicans*, *C. auris*, and *C. neoformans* following the EUCAST guidelines. Overnight cultured cells were spun down and washed three times with phosphate buffered saline (PBS) buffer, and the cell number was synchronized as in OD_600_ value of 1.0 as a fungal cell stock solution. A final 1/100 dilution in RPMI 1640 medium (Sigma-Aldrich #R6504) was separated into 96-well plates for the assay. Each compound was dissolved in dimethyl sulfoxide (DMSO) to a final concentration was 5.12 mg/ml as a drug stock solution. For the assay, 5 μl of the drug stock was inoculated into 195 μl of the fungal cell stock solution and serially diluted into the 100 μg/ml of the fungal cell stock solution for 2-fold dilution. The final drug concentration for MIC assay ranged from 1 μg/ml to 128 μg/ml. The plates were incubated at 35°C standing incubator for 48 h, and OD_600_ value was detected by a microplate reader. Enterobactin from *Escherichia coli* (E3910, Sigma-Aldrich, Merch) was used as a structural related control to the compounds.

### Gene expression analysis

*C. albicans* was treated with compounds, followed by analysis of gene expression, particularly of genes related to drug responses and virulence factors, as previously described (Lee et al., 2022). Overnight-grown cells were inoculated into the fresh YPD broth at an OD_600_ value of 0.2 and sub-cultured to 0.8 in the early exponential growth phage. Each compound was added to the cultured broth at of 50% inhibitory concentration in Table 2, with a maximum concentration of 128 μg/ml. After 3 h of treatment, cells were spun down, and the total RNA was extracted using RNAiso PLUS (TaKaRa #9108) with beads beating. The extracted RNA used as a template to synthesize cDNA, and the expression was detected by real-time quantitative PCR (TaKaRa, CronoSTAR 96 Real-Time PCR System). Primers used to detect the gene expression are listed in Table 3.

### Anti-candidalysin antibody production

A custom polyclonal antibody against candidalysin was generated by GenScript (China) using a synthetic peptide corresponding to a partial C-terminal amino acid sequence of the candidalysin toxin. The peptide was synthesized and conjugated to keyhole limpet hemocyanin (KLH) to enhance immunogenicity. Two New Zealand White rabbits were immunized subcutaneously with the KLH-conjugated peptide emulsified in Freund’s adjuvant, followed by multiple booster injections according to the manufacturer's protocol. Serum was collected and affinity-purified using a peptide-conjugated affinity column.

### Indirect ELISA for candidalysin detection

To quantify candidalysin levels in culture supernatants, an indirect Enzyme-Linked Immunosorbent Assay (ELISA) was performed using the anti-candidalysin antibody described above. *C. albicans* cells were cultured in RPMI 1640 medium with or without compound treatment for 3 h at 35°C. The culture supernatants were collected by centrifugation (10,000 × g, 10 min), and the supernatants were subsequently concentrated using Amicon Ultra-15 centrifugal filter units (3 kDa MWCO; Merck Millipore) according to the manufacturer’s instructions. The total peptide concentration in the concentrated supernatants was measured using the BCA protein assay kit (Thermo Fisher Scientific). The concentrated supernatants were used as the antigen source and coated onto 96-well ELISA plates (Germany) using Carbonate Coating Buffer B (Invitrogen, Thermo Fisher Scientific) at 4°C overnight. After blocking with 1% BSA in PBST (PBS with 0.05% Tween-20), the wells were incubated with the anti-candidalysin antibody (1:1,000 dilution in PBST), followed by incubation with an HRP-conjugated anti-rabbit IgG secondary antibody (1:2,000 dilution). TMB substrate was used for color development, and absorbance was measured at 450 nm using a microplate reader (Molecular Devices).

### In vivo efficacy analysis

For in vivo antifungal efficacy testing, overnight cultured *C. albicans* cells were subcutaneously inoculated into mice according to previously reported methods with slight modifications (Choi et al., 2021). SPF/VAF-confirmed inbred 6-week-old female BALB/cAnNCrlOri mice were purchased from ORIENT BIO INC. (Korea) and acclimatized to the breeding environment for one week before the experiment. The mice were anesthetized with isoflurane, and the hair on their back was shaved with a trimmer. The number of yeast cells was adjusted to 10^8^ cells/ml in PBS buffer, and the 100 μl was infected subcutaneously. The condition of the mice was monitored daily and a final 1% aqueous solution of EtOH dissolved each compound was applied daily to the injection site. For the fungal burden assay, the abscesses of the sacrificed mice were acquired, weighted, homogenized, and spread onto YPD plates to incubate the fungal cells. The statistical significance of the difference was determined using one-way ANOVA with Tukey’s multiple-comparison test. GraphPad Prism 9.5.1 was used for statistical analysis.

### Protein structure preparation and molecular docking

Due to the lack of experimentally determined three-dimensional structures in the Protein Data Bank (PDB) for the target transcription factors, protein models were predicted using AlphaFold3 (Abramson et al., 2024). Potential *ECE1* promoter-binding transcription factors from *C. albicans* (Ahr1, Bcr1, Brg1, Efg1, Fkh2, Mcm1, Ndt80, Nrg1, and Ume6) were modeled using the AlphaFold3 server. However, manual inspection of the predicted structures, along with pLDDT scores, indicated that models for Ahr1, Fkh2, and Ndt80 show sufficient confidence for further analysis, while the others were not suitable for further analysis due to very low pLDDT scores. Therefore, molecular docking experiments were performed using the AlphaFold3 models of Ahr1, Fkh2, and Ndt80. Prior to docking, residues predicted with low confidence (pLDDT < 50) were trimmed from the models. The structures of the ligands (compounds 1–4) were generated using Phenix eLBOW (Liebschner et al., 2019; Moriarty et al., 2009). Molecular docking was performed using Autodock Vina via the dockingpie extension within Pymol (Schrödinger)(Eberhardt et al., 2021; Rosignoli and Paiardini, 2022). Protein and ligand preparation steps required for docking were handled using the tools within this workflow. The docking results indicated that the predicted binding energies for compounds 1–4 to each of the selected transcription factor models did not show significant differences.

### Statistical analysis

All data are presented as mean ± standard error of the mean (SEM) from three independent biological replicates. Statistical analyses were performed using GraphPad Prism version 10. One-way ANOVA followed by Tukey’s post hoc test was used for multiple group comparisons. A *P*-value < 0.05 was considered statistically significant. Asterisks indicating statistical significance were applied according to the following criteria: *P* < 0.05 (*), *P* < 0.01 (**), and *P* < 0.001 (***).

## Results

### Isolation and NMR-based characterization of compound 1

**Fig. 1.**
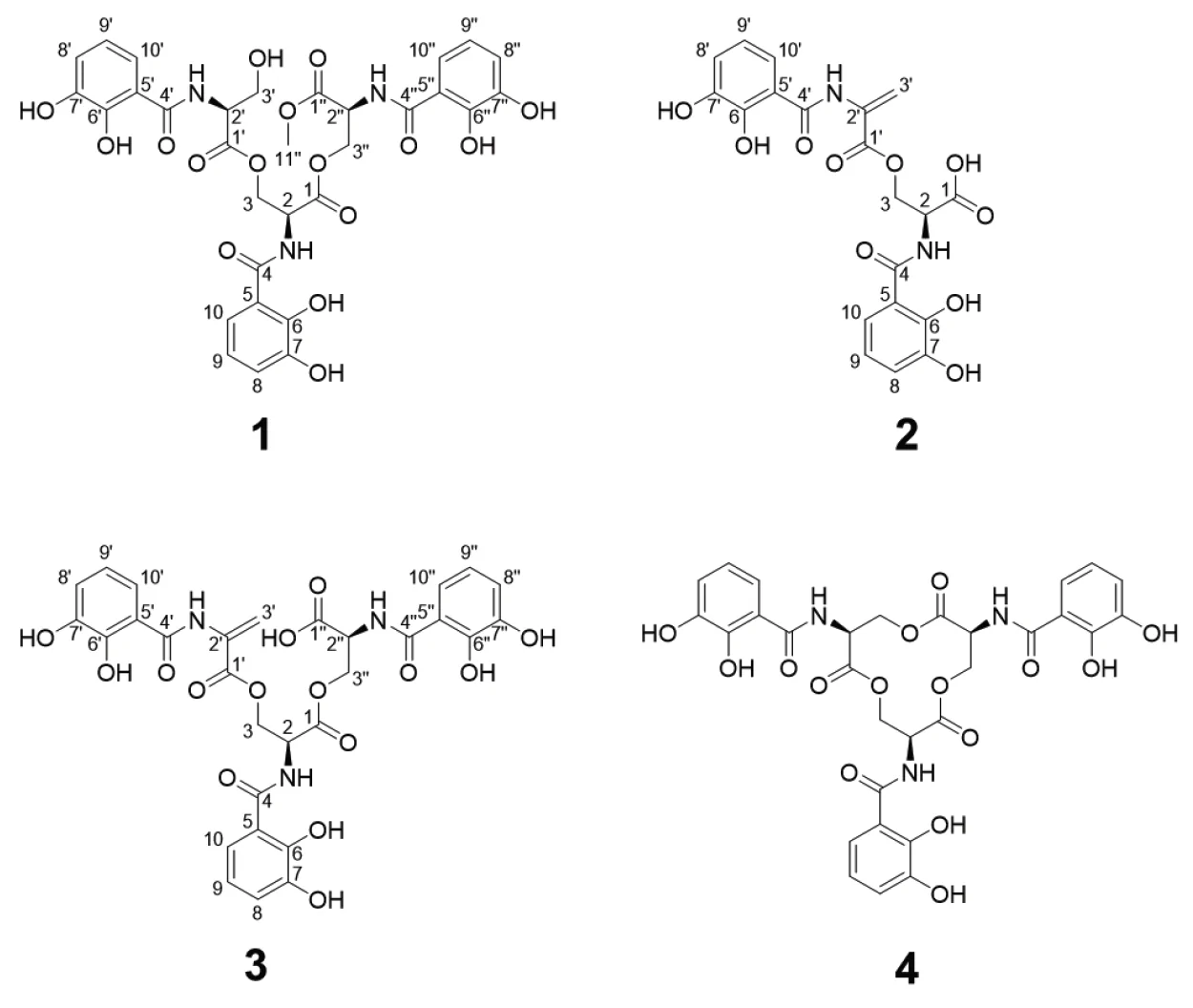
Structure of methoxy-apo-enterobactin (1), *N*-(2,3-dihydroxybenzoyl)-l-serine 3-[2-[(2,3-dihydroxybenzoyl)amino]-2-propenoate] (2), *N*, *N*′, *N*′′-Tris(2,3-dihydroxybenzoyl)-*O*-(*α*-aminoacryloyl)-*O*-serylserine (3), and Enterobactin (4).

**Fig. 2.**
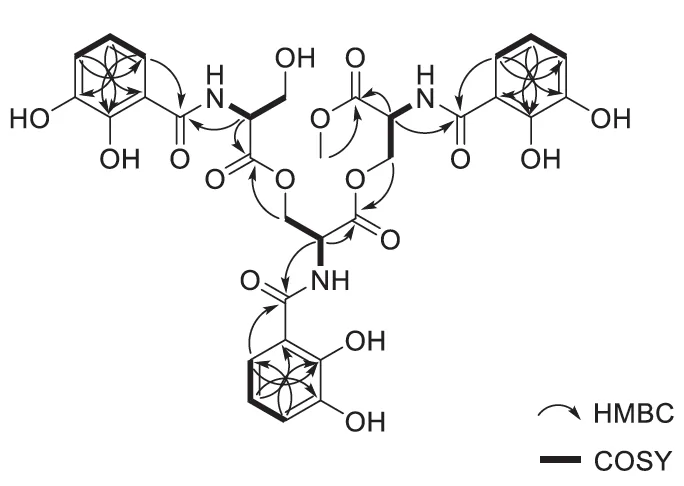
Key COSY and HMBC correlations of methoxy-apo-enterobactin.

Methoxy-apo-enterobactin (1) was obtained as a brown oil, and its molecular formula was determined to be C_31_H_31_N_3_O_16_ (*m/z* [M+H]^+^ 702.1769 (calculated for C_31_H_32_N_3_O_16_, 702.1777) by HR-ESI-MS analysis with ^1^H and ^13^C NMR data (Table 1). The ^1^H and HSQC NMR spectra of 1 in dimethyl sulfoxide-*d*_6_ (DMSO-*d*_6_) showed nine benzyl side chain protons which resonate between 6.69 and 7.37 ppm (δ_H_ 7.37, 7.32, 7.30, 6.94 [3H], and 6.69 [3H]), three methine protons (δ_H_ 4.88 [2H] and 4.58), six aliphatic protons (δ_H_ 4.59, 4.57, 4.46, 4.43, 3.82, and 3.76), and one *O*-methyl group protons (δ_H_ 3.62 [3H]). Analysis of the ^13^C NMR spectrum along with the HSQC spectrum indicated that 1 has six carbonyl carbons (δ_C_ 169.5, 169.4, 169.1 [2C], 168.6, and 168.4), eighteen phenyl group carbons (δ_C_ 149.0 [2C], 148.3, 146.3 [2C], 146.0, 119.1, 118.9, 118.8, 118.6, 118.3 [2C], 118.2 [3C], 116.0, and 115.4 [2C]), three α-amino carbons (δ_C_ 51.5, 51.4, and 55.2), three methylene bridge carbons (δ_C_ 63.6, 63.1, and 60.8), and one *O*-methyl carbon (δ_C_ 52.4). By comparing the UV spectrum of 1 with in house UV library, it was suggested that compound 1 was structurally related to enterobactin (4)(Fiedler et al., 2001) (Fig. 1). Through 2D NMR analysis, including HSQC, COSY, and HMBC, the planar structure of 1 was determined to bear three moieties: two *N*-(2,3-dihydroxybenzoyl)-serine and one *N*-(2,3-dihydroxybenzoyl)-serine methyl ester (Figs. 1 and 2). The HMBC correlations from H-8 to C-6 (δ_C_ 149.0), H-9 to C-7 (δ_C_ 146.3) and C-5 (δ_C_ 115.4), and H-10 to C-6 indicated the 2,3-dihydroxybenzene ring. The ring moiety was also supported by the COSY correlations between H-9 (δ_H_ 6.69)/H-8 (δ_H_ 6.96) and H-9/H-10 (δ_H_ 7.83). The serine residue was also determined by a combined analysis of the COSY and HMBC data, which showed the COSY correlation between H-2 (δ_H_ 4.89) and H2-3 (δ_H_ 4.58, 4.44) and the HMBC correlation H-2 to C-1 (δ_C_ 168.6). Additionally, the connectivity between serine and 2,3-dihydroxybenzene ring was confirmed by HMBC correlation of H-2 to C-4 (δ_C_ 169.1). Along similar lines, three *N*-(2,3-dihydroxybenzoyl)-serine were identified. Among them, the characteristic HMBC signal of the *O*-methyl proton, H-11'' (δ_H_ 3.62), connected H-11'' and C-1'' (δ_C_ 169.5). Thus, two *N*-(2,3-dihydroxybenzoyl)-serines and *N*-(2,3-dihydroxybenzoyl)-serine methyl ester were established. Lastly, the HMBC correlations from H-3 to C-1' (δ_C_ 169.4) and H-3'' to C-1 constructed the linkages between these three moieties, with this, the planar structure of compound 1 was established. The absolute configuration of the serine residues in 1 was determined to be L using advanced Marfey's methods.

### Antifungal efficacy of compounds against human fungal pathogens

To evaluate the antifungal efficacy of the isolated compounds, we performed minimum inhibitory concentration (MIC) assay against major invasive fungal pathogens including *C. albicans*, *C. auris*, and *C. neoformans*. Among the compounds from *S. ambofaciens* CJD34, compound 1 exhibited inhibitory efficacy against *C. albicans* with an IC_50_ of 16 μg/ml. Compound 3 inhibited the *C. albicans* growth by approximately 20% at a concentration of 32 μg/ml, while enterobactin (4) showed IC_20_ of 8 μg/ml. Consequently, these results indicate that compound 1 exhibited narrow-spectrum antifungal activity, selectively inhibiting the growth of *C. albicans*.

### Compound 1 inhibited the expression of *ECE1* encoding candidalysin

**Fig. 3.**
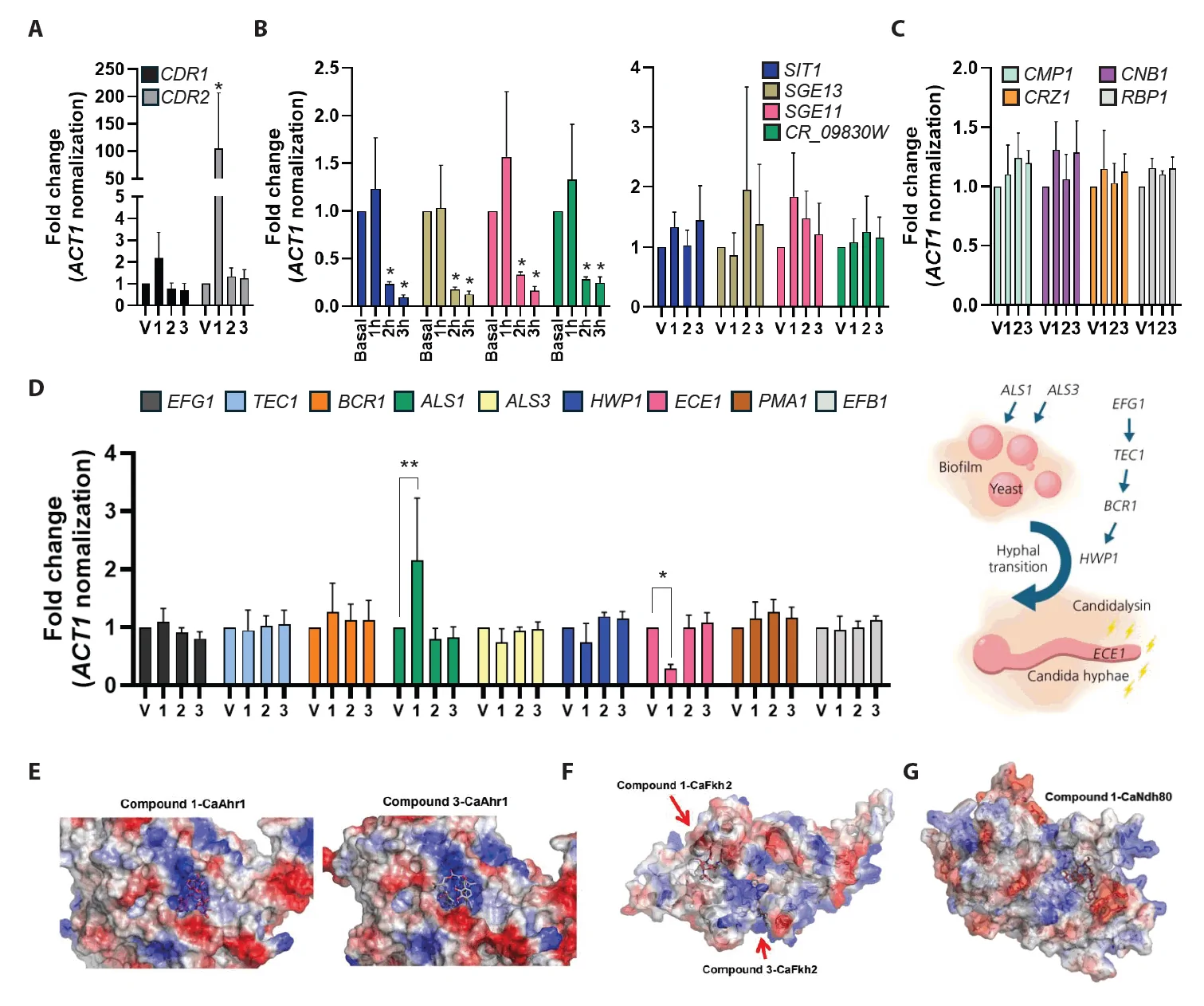
Specific inhibition of *ECE1* expression by compound 1. (A–D) Gene expression analysis under specific conditions in *Candida albicans*. Cells in early-exponential phase were treated with each drug at the IC_50_ concentration and incubated for 3 h. Total RNA was extracted, reverse-transcribed into cDNA, and analyzed by quantitative PCR. (A) Expression of drug efflux pump genes. (B) Expression of siderophore transporter genes following treatment with enterobactin (basal, and each time-point) or with each compound. (C) Expression of genes in calcineurin pathway. (D) Expression of genes associated with biofilm formation and hyphal transition in *C. albicans*. (E–G) Prediction of compound-protein interactions (CPIs). Using AlphaFold3-based structural modeling, protein structures of *C. albicans* transcription factors were generated, and docking simulations were performed with compounds isolated from *Streptomyces ambofaciens* CJD34. (E) Docking model of compound 1 or 3 with the *C. albicans* transcription factor Ahr1. (F) Docking model of compound 1 with Fkh2. (G) Docking model of compound 1 with Ndh80.

To investigate the potential targets of compounds, *C. albicans* cells were treated with the compounds, and the expression of the genes related to drug responses and virulence factors was analyzed. We first examined the expression of drug efflux pumps *CDR1* and *CDR2*. Interestingly, treatment with compound 1 resulted in a 2-fold upregulation of *CDR1* expression and a greater than 2-fold increase in *CDR2* expression (Fig. 3A). These results indicate that compounds 1 triggers transcriptional activation of *CDR1* and *CDR2*, consistent with a typical drug response (Maheronnaghsh et al., 2022).

We next analyzed the expression of siderophore transporters, which are closely associated with iron uptake and siderophores biosynthesis in fungi (Pecoraro et al., 2021). Specifically, the expression levels of *SIT1*, *SGE11*, *SGE13*, and *CR_09830W* were assessed following treatment with enterobactin (4), which served as a structurally related control for comparison with compounds. After 3 h exposure, the expression of these iron acquisition-related genes was markedly reduced. In contrast, the treatment with each compound did not significantly alter the expression of siderophore transporter genes (Fig. 3B). Notably, the expression of genes of the calcineurin signaling pathway, a major regulator of fungal stress adaptation and virulence, remained unaffected by compound 1 treatment (Fig. 3C). Subsequently, we examined the expression of the genes related to yeast-to-hyphal transition and biofilm formation, which are the key virulence traits of *C. albicans*. Compound 1 induced the expression of *ALS1*, a gene associated with biofilm formation, while suppressing *ECE1*, the gene encoding candidalysin (Fig. 3D). These findings suggest that compound 1 selectively targets *ECE1* without broadly impacting general stress response pathways.

To further explore the mechanism of *ECE1* repression, we evaluated the potential binding of compound 1 to known *ECE1* transcriptional regulators, including Ahr1, Bcr1, Brg1, Efg1, Fkh2, Mcm1, Ndt80, Nrg1, and Ume6. Among these, Ahr1 is particularly critical for the expression of both *ECE1* and *ALS3* (Ruben et al., 2020). Molecular docking analyses predicted that compound 1 displayed either weak or comparable binding affinities to those observed for compounds 3 (Fig. 3E, 3F, and 3G). These results suggest that the repression of *ECE1* expression by compound 1 is unlikely to result from direct binding to upstream transcription factors.

Collectively, these findings demonstrate that compound 1 specifically inhibits *ECE1* expression, leading to suppression of candidalysin production, without affecting broader stress adaptation or iron sensing pathways in *C. albicans*.

### Compound 1 reduced candidalysin production

**Fig. 4.**
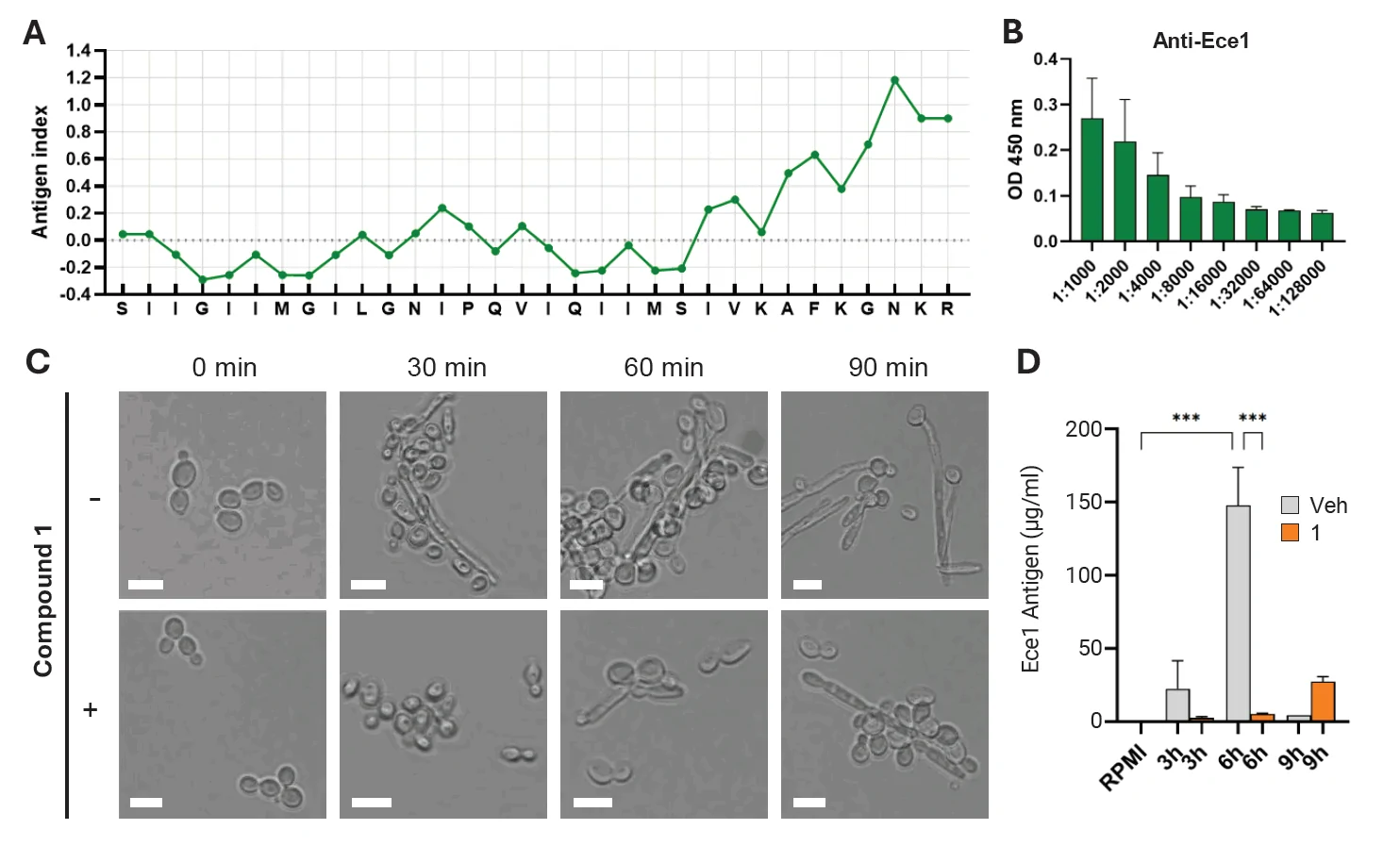
Quantitative assessment of Ece1 inhibition by compound 1. (A) Jameson-Wolf antigenic index prediction of the Ece1-derived peptide. Antigenicity was calculated based on a weighted combination of hydrophilicity, flexibility, and surface accessibility. (B) ELISA detection of anti-Ece1 antibody response, showing reactivity against the 4 μg of synthetic Ece1 peptide. Antibody levels were measured as OD450 values. (C) Cell morphology after treatment with compound 1. Microscopic images illustrate structural changes and hyphal development under treated versus untreated conditions. (D) Quantification of Ece1 antigen (μg/ml) in culture supernatants following compound 1 treatment. Antigen concentrations were determined using a standard-curve-based ELISA. A decrease in Ece1 levels indicates compound-mediated inhibition of secretion or expression.

To further validate the inhibitory effect of compound 1 on candidalysin production, an indirect ELISA was performed using a custom-made anti-candidalysin antibody. The antibody was generated against a partial C-terminal amino acid sequence of the candidalysin peptide. Using this antibody, candidalysin secreted into the culture supernatant was successfully detected (Fig. 4). Given that *C. albicans* cells treated with compound 1 exhibited a modest delay in the yeast-to-hyphal transition (Fig. 4C), we hypothesized that candidalysin secretion would also be delayed. Indeed, culture supernatants collected up to 6 h after treatment showed a significant reduction in candidalysin levels compared to the untreated control. Although candidalysin production eventually restored by 9 h post-treatment, the initial suppression indicates that compound 1 delays, rather than completely inhibits, candidalysin secretion (Fig. 4D). These findings suggest that compound 1 transiently suppresses candidalysin production by delaying hyphal development and *ECE1* expression.

### Compound 1 treatment reduced fungal burden in murine subcutaneous *C. albicans* infection model

**Fig. 5.**
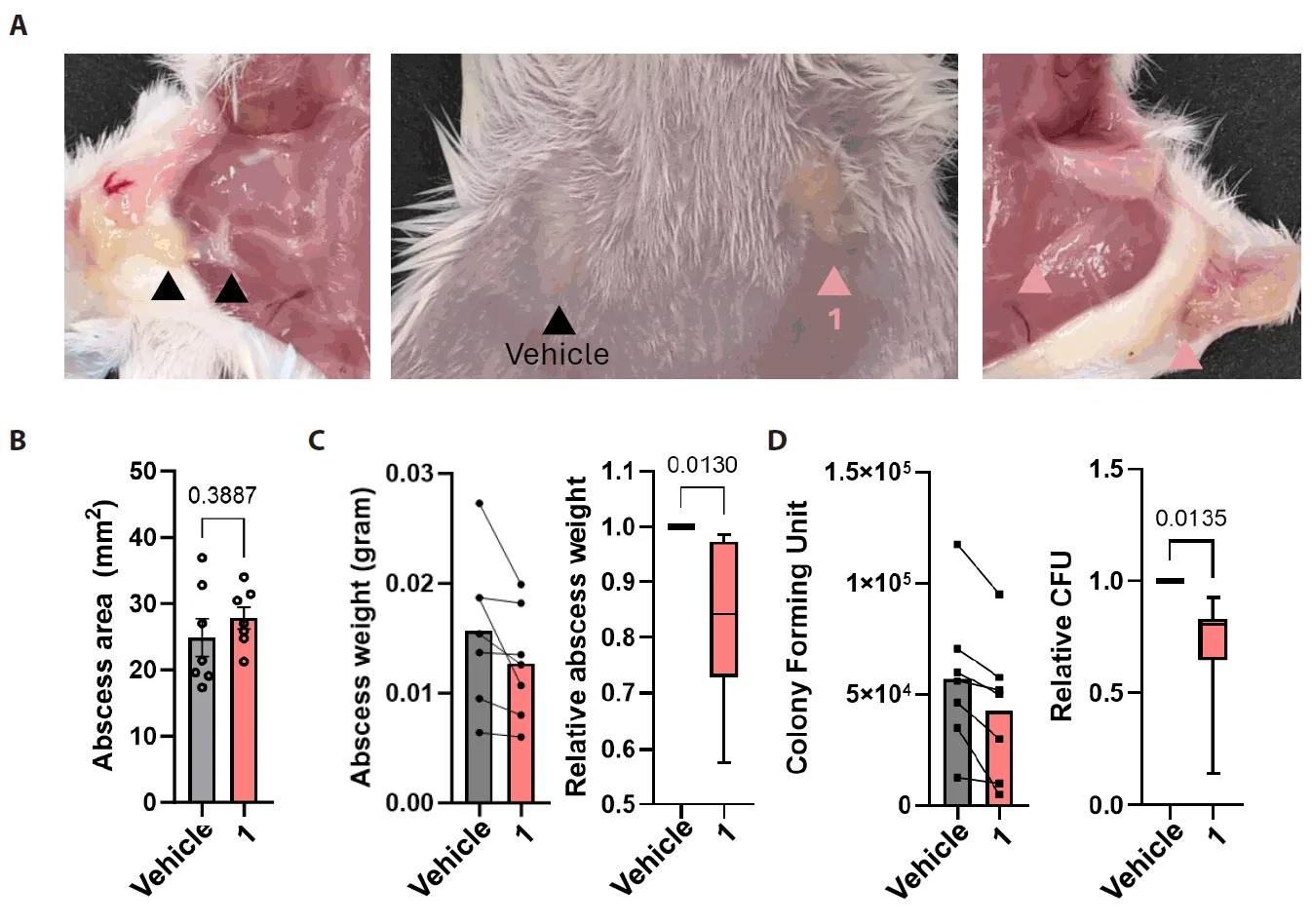
In vivo efficacy of compound 1 against subcutaneous candidiasis. *Candida albicans* cells (10^7^) were subcutaneously injected into the buttocks of 7-week-old Balb/c mice (n = 7). After infection, either the vehicle or each drug was topically applied to the skin for 10 days. (A) The size of the abscess formed on the buttocks of sacrificed mice was compared, and surgical confirmation was conducted to assess the degree of infection. (B–D) The quantitative area of the abscess formed at the infected site was measured (B), and a fungal burden assay was performed. Abscess samples were homogenized (C), spread on yeast-extracted-peptone agar plates to quantify colony-forming units (CFUs) (D).

We next evaluated the in vivo efficacy of compound 1 using a murine model of subcutaneous *Candida albicans* infection. The subcutaneous infection model is a widely used system for assessing the topical antifungal activity of drug candidates (Gil et al., 2022). In this model, fungal inoculation resulted in visible swelling and abscess formation at the infected sites. To enable direct comparison within the same animal, the left and right infection areas of each mouse were treated with either vehicle or the compound 1, respectively (Fig. 5A). Although the surface area of infection did not significantly differ between treatment groups (Fig. 5B), the abscess excised from compound 1-treated sites were visibly smaller and weighted less than those from the vehicle-treated group (Fig. 5C). Quantitative fungal burden analysis further demonstrated a reduction in the number of fungal cells in compound 1-treated abscesses compared to controls (Fig. 5D). These results suggest that compound 1 has potential as a topical treatment option for subcutaneous candidiasis.

## Discussion

*Candida albicans* is a major opportunistic fungal pathogen, particularly dangerous to immunocompromised individuals, and its pathogenicity is largely attributed to the yeast-to-hyphal transition and the secretion of virulence factors such as candidalysin (Moyes et al., 2016). Candidalysin, a peptide toxin encoded by the *ECE1* gene and processed by Kex2 and Kex1 proteases, plays a crucial role in epithelial damage and immune activation (Moyes et al., 2015). Therefore, targeting candidalysin production presents a promising antifungal therapeutic strategy that focuses on suppressing virulence rather than fungal viability.

In this study, we identified methoxy-apo-enterobactin (compound 1), a natural siderophore-like molecule produced by *Streptomyces ambofaciens* CJD34, as a potent inhibitor of candidalysin expression and secretion. Transcription analysis revealed that compound 1 significantly downregulates *ECE1* expression in *C. albicans* without affecting the calcineurin or iron-sensing pathways, suggesting a degree of specificity in its mechanism of action. Furthermore, treatment with compound 1 induced the expression of *CDR1* and *CDR2*, genes encoding ATP-binding cassette (ABC) transporters involved in xenobiotic efflux. This indicates that *C. albicans* may recognize compound 1 as a foreign chemical and activate a basal protective response. However, the selective inhibition of *ECE1* expression was not driven by a generalized stress response. Molecular docking further supported this by showing only weak or non-specific interactions with known *ECE1* transcription factors. ELISA-based detection confirmed that candidalysin secretion into the culture supernatant was markedly delayed upon treatment with compound 1 treatment. These functional validations corroborate the transcriptional data, and suggest that methoxy-apo-enterobactin specifically modulates the transcription and expression of candidalysin.

In a murine subcutaneous infection model, topical application of compound 1 led to a significant reduction in fungal burden, despite minimal changes in lesion size. This in vivo efficacy highlights the potential of compound 1 as a topical therapeutic agent for subcutaneous candidiasis. The reduced fungal burden was consistent with impaired virulence, likely resulting from diminished candidalysin production and altered host-pathogen interaction dynamics. However, several limitations remain. The efficacy of compound 1 in systemic candidiasis models has yet to be evaluated. Additionally, beyond fungal virulence attenuation, the potential effects of compound 1 on host immune responses require further investigation. Future studies should address these limitations by evaluating systemic pharmacokinetics, optimizing compound stability and delivery systems, and exploring broader applications against invasive fungal infections.

Although compound 1 shares structural similarity with enterobactin, it exhibited distinct biological effects. Enterobactin, primarily produced by Gram-negative bacteria, strongly chelates Fe(III) ions through hexadentate coordination and is transported via specific siderophore uptake systems to maintain iron homeostasis (Raymond et al., 2003). The enterobactin (designated as compound 4 in Fig. 1) used in this study was purified from *E. coli* and is classified as a catecholate-type siderophore. In fungi, siderophores are predominantly hydroxamate-type, and although it has been rarely reported, in *Saccharomyces cerevisiae* can import enterobactin via transporters belonging to the major facilitator superfamily, including Enb1p [see review by (Choi et al., 2024)]. In our study, enterobactin treatment resulted in up to 20% inhibition of *C. albicans* growth at a concentration of 8 μg/ml and led to the downregulation of siderophore transporter gene expression (Fig. 3B). This transcriptional repression is likely due to altered environmental iron sensing, regardless of whether direct siderophore uptake occurred. In contrast, compound 1, despite its structural similarity to enterobactin, did not affect the expression of siderophore transporter genes, highlighting its distinct mode of action.

In the structure of enterobactin, Fe(III) is centrally coordinated and stabilized by six oxygen atoms from the three catecholate arms through hexadentate chelation. This highly stable coordination enables enterobactin to efficiently sequester and transport iron, contributing to its exceptional ferric ion affinity (Abergel et al., 2006). In contrast, the structures of compound 1 and compound 3, in which the central triserine-derived macrocyclic ring of enterobactin is opened, exhibit a reduced number or accessibility of donor oxygen atoms for Fe(III) coordination. This structural modification is predicted to impair the formation of stable hexadentate complexes with ferric ions. In particular, compound 1, a methoxy-substituted derivative of the catecholate moiety, is likely to exhibit significantly lower iron chelation capacity compared to enterobactin due to both steric hindrance and decreased electron-donating ability. For these reasons, unlike the structurally related enterobactin, compound 1 does not interfere with fungal iron sensing or mimic an iron-rich environment, strongly suggesting that it inhibits candidalysin production independently of siderophore-mediated iron signaling.

Overall, our findings demonstrate that methoxy-apo-enterobactin functions as a novel inhibitor of candidalysin-mediated virulence. Although compound 1 exhibits weaker direct fungicidal activity compared to conventional antifungal agents, it was confirmed to attenuate pathogenicity by suppressing virulence factor production. These results support the emerging paradigm of antivirulence strategies in antifungal drug development and provides a promising candidate for further preclinical development.

## Figures and Tables

**Table 1.** NMR spectroscopic data for compounds 1–3

Position	Methoxy-apo-enterobactin (1)^a^		*N*-(2,3-dihydroxybenzoyl)-l-serine 3-[2-[(2,3-dihydroxybenzoyl)amino]-2-propenoate] (2)^a^		*N*,* N′*, *N′′*-Tris(2,3-dihydroxybenzoyl)-*O*-(*α*-aminoacryloyl)-*O*-serylserine (3)^a^	
	δ_C_, type	δ_H_, mult (*J* in Hz)	δ_C_, type	δ_H_, mult (*J* in Hz)	δ_C_, type	δ_H_, mult (*J* in Hz)
1	168.6, C	-	170.3, C	-	171.6, C	-
2	51.5, CH	4.89, m	51.5, CH	4.88, m	52.3, CH	4.65, m
3	63.1, CH_2_	4.58, m	64.5, CH_2_	4.63, dd (11.0, 7.0)	64.6, CH_2_	4.62, m
		4.44, m		4.59, dd (11.0, 7.0)		4.44, m
4	169.1, C	-	168.7, C	-	168.5, C	-
5	115.4, C	-	115.7, C	-	116.0, C	-
6	146.3, C	-	148.6, C	-	147.7, C	-
7	149.0, C	-	146.2, C	-	146.1, C	-
8	119.1, CH	6.96, m	118.9, CH	6.94, d (7.5)	118.4, CH	6.92, m
9	118.2, CH	6.69, m	118.3, CH	6.71, t (8.0)	117.8, CH	6.67, m
10	118.3, CH	7.33, d (8.0)	118.2, CH	7.35, d (8.0)	120.3, CH	7.37, d (8.0)
NH		9.23, s		9.21, d (6.5)		
1'	169.4, C		163.2, C	-	162.9 C	-
2'	55.3, CH	4.58, m	132.1, C	-	132.3, C	-
3'	60.8, CH_2_	3.82, m	109.3, CH_2_	6.54, s	108.8, CH_2_	6.54, s
		3.76, dd (11.0, 4.0)		5.81, s		5.75, s
4'	168.4, C	-	164.7, C	-	168.9, C	-
5'	116.0, C	-	118.5, C	-	119.1, C	-
6'	148.3, C	-	145.8, C	-	148.6, C	-
7'	146.0, C	-	146.1, C	-	147.1, C	-
8'	118.8, CH	6.96, m	118.6, CH	6.99, d (7.5)	118.4 CH	6.92, m
9'	118.2, CH	6.69, m	118.9, CH	6.75, t (8.0)	117.8, CH	6.67, m
10'	118.6, CH	7.38, d (8.0)	120.2, CH	7.38, d (8.0)	118.1, CH	7.30, d (8.0)
NH		8.97, s		10.7, s		7.85, m
1''	169.5, C	-	-	-	168.7, C	-
2''	51.4, CH	4.86, m	-	-	51.5, CH	4.99, m
3''	63.6, CH_2_	4.57, m	-	-	64.2, CH_2_	4.62, m
		4.46, m		-		4.44, m
4''	169.5, C	-	-	-	164.9, C	-
5''	115.4, C	-	-	-	115.5, C	-
6''	149.0, C	-	-	-	149.6, C	-
7''	146.3, C	-	-	-	146.6, C	-
8''	118.9, CH	6.96, m	-	-	118.4, CH	6.92, m
9''	118.2, CH	6.69, m	-	-	117.4, CH	6.61, t (8.0)
10''	118.3, CH	7.30, d (8.0)	-	-	118.1, CH	7.33, d (8.0)
11''	52.4, CH_3_	3.62, s	-	-	-	-
NH		9.22, s	-	-	-	-

^1^H and ^13^C NMR spectroscopic data for Methoxy-apo-enterobactin (1), *N*-(2,3-dihydroxybenzoyl)-L-serine 3-[2-[(2,3-dihydroxybenzoyl)amino]-2-propenoate] (2), and *N*, *N*′, *N*′′-Tris(2,3-dihydroxybenzoyl)-*O*-(*α*-aminoacryloyl)-*O*-serylserine (3) in DMSO-*d*_6_

a ^1^H and ^13^C NMR data were recorded at 700 and 175 MHz respectively.

**Table 2.** Antifungal activity of the compounds against human fungal pathogens

Cpd.	*C. albicans*			*C. auris*			*C. neoformans*		
	IC_20_	IC_50_	IC_90_	IC_20_	IC_50_	IC_90_	IC_20_	IC_50_	IC_90_
1	8	16	>128	>128	>128	>128	128	>128	>128
2	>128	>128	>128	>128	>128	>128	>128	>128	>128
3	32	>128	>128	>128	>128	>128	>128	>128	>128
4	8	>128	>128	>128	>128	>128	>128	>128	>128

* IC_20_, IC_50_, and IC_90_ refer to 20%, 50%, and 90% inhibitory concentrations (μg/ml) by MIC assay.

**Table 3.** Primers used in this study

Name	Target	Sequence (5’ to 3’)
P00074	*CDR1 *qLP	CCAATTATTTGGCTGGTGCT
P00075	*CDR1* qRP	GCCAAAAAGAATACGGCAAA
P00076	*CDR2* qLP	CAATTTTGTCGCAATTGGTG
P00077	*CDR2* qRP	GTGCTCCACCTCGGAAATAA
P00078	*EFG1* qLP	CCCCCATACCTTCCAATTCT
P00079	*EFG1* qRP	TTGGTTGTTGCATTGTCGAT
P00080	*TEC1* qLP	AAATATTGCTCCCGCAGTTG
P00081	*TEC1* qRP	TATGTGTGGGTGATGCGTTT
P00082	*BCR1* qLP	CAGCACGCATCTATGGCTTA
P00083	*BCR1* qRP	AGGTATTGGTGGCAATGGAG
P00084	*ALS1* qLP	CCTGCTGGTTATCGTCCATT
P00085	*ALS1* qRP	GACGACTGCCAGCACAAGTA
P00086	*ALS3* qLP	TTGCGACTGCAAAGACAATC
P00087	*ALS3* qRP	CCCAAAACAGCATTCCAAGT
P00088	*HWP1* qLP	TCAGTTCCACTCATGCAACC
P00089	*HWP1* qRP	GCAGCACCGAAAGTCAATCT
P00090	*ECE1* qLP	AGCTGTTGACACAGCCATGA
P00091	*ECE1* qRP	CTTGGCATTTTCGATGGATT
P00092	*PMA1* qLP	TCCAACCTTTCGATCCTGTC
P00093	*PMA1* qRP	TTGGGTGGTCATCTTCAACA
P00094	*EFB1* qLP	TCACCTTGGATGTCAAACCA
P00095	*EFB1* qRP	ACTGGAATCCATTGGTGAGC
P00096	*ACT1* qLP	CAGTTTTGTTGACCGAAGCT
P00097	*ACT1* qRP	GAAACGTAGAAAGCTGGAAC
P00192	*CMP1* qLP	GGAGCAACACCAGTATCACC
P00193	*CMP1* qRP	GACTTCTCTTCTTCGTTGGCTC
P00194	*CNB1* qLP	GGAAGCTGATTTGGACGGTG
P00195	*CNB1* qRP	TGTTGGCAATTGTATCGGTGT
P00196	*CRZ1* qLP	TGTGAGTACTGCAGCTACGA
P00197	*CRZ1* qRP	ACCTTGCTCTGTCCGTTGTA
P00198	*RBP1* qLP	TGCTAAGCCAGGTGATACAGT
P00199	*RBP1* qRP	ACTTGACCAACACCAACAGT
P00373	*SIT1* qLP	TGTCAGTGCCCAAAGTTGTG
P00374	*SIT1* qRP	ACCTGCTACTGACGAACCAA
P00375	*SGE13* qLP	TTCATGGTGCCGATGTTGTC
P00376	*SGE13* qRP	GGCCACCCATTTCTGAAGTT
P00377	*SGE11* qLP	ACCATCGACGCCTCAAAATG
P00378	*SGE11* qRP	TCCCTTTTGTTCTGCCGTTG
P00379	CR_09830W qLP	AACAATGGTGGCTGCACAAA
P00380	CR_09830W qRP	CTAAACCAACAGCACCACCC
